# SHINE_color: Controlling low-level properties of colorful images

**DOI:** 10.1016/j.mex.2023.102377

**Published:** 2023-09-16

**Authors:** Rodrigo Dal Ben

**Affiliations:** Ambrose University, 150 Ambrose Circle SW, Calgary, AB T3H 0L5, Canada

**Keywords:** Visual perception, Luminance control, Image manipulation, SHINE_color

## Abstract

Visual perception combines top-down processes arising from participants individual histories, such as expectations and goals, and bottom-up processes that arise from visual stimuli properties, such as luminance and contrast. The precise control of low-level visual stimuli properties is essential when investigating visual perception. Without it, for instance, investigations of bottom-up processes are virtually impossible and investigations of top-down processes risk major confounds when testing and formulating hypotheses. The SHINE (spectrum, histogram, and intensity normalization and equalization) toolbox for MATLAB [1] allows precise control of images’ Fourier amplitude spectra, the normalizing and scaling of luminance and contrast, and exact histogram specification optimized for perceptual visual quality. Following Willenbockel and Cols (2010) advices, here we present an adaptation of the SHINE toolbox, named SHINE_color, which extends SHINE functionalities by allowing the parametrical manipulation of low-level properties of both static and animated colorful images.

• Parametric manipulation of low-level properties of colorful images
• Spectrum, histogram, and intensity normalization and equalization

## Graphical abstract

**Figure:**
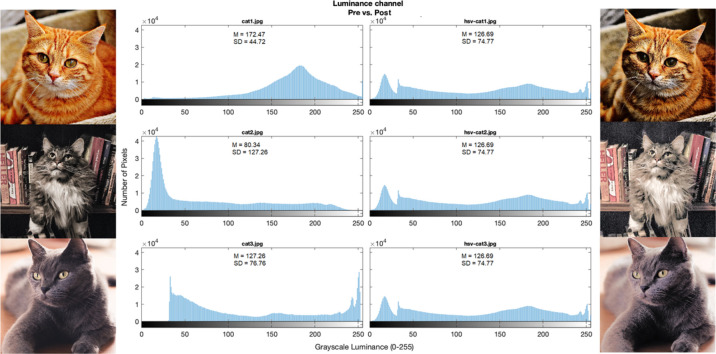

Specifications table

**Table:** 

Subject area:	Psychology
More specific subject area:	Perception and Sensation
Name of your method:	SHINE_color
Name and reference of original method:	Willenbockel, Verena, Javid Sadr, Daniel Fiset, Greg O. Horne, Frédéric Gosselin, and James W. Tanaka. 2010. Controlling low-level image properties: The SHINE toolbox. Behavior Research Methods, 42 (3): 671–84. doi:10.3758/BRM.42.3.671.
Resource availability:	Supporting information: https://osf.io/xk8bt/ Required software: https://www.mathworks.com/

## Method details

### Statement of need

One powerful way to control low-level properties of visual stimuli is to use the SHINE (spectrum, histogram, and intensity normalization and equalization) toolbox for MATLAB [1]. This toolbox builds on MATLAB image processing toolbox and contains a set of functions that allows the parametric specification of luminance and contrast, histogram, and Fourier amplitude spectra of static images. By doing so, it minimizes potential low-level confounds when investigating higher-level processes (e.g., cognitive effort, recognition). However, SHINE only works with greyscale images. Whereas this serves well to many research purposes (e.g., [2,3]), other research requires colorful stimuli (e.g., [4,5,6]). Here, we describe the SHINE_color, an adaptation of SHINE that allow users to perform all operations from SHINE toolbox on both static and dynamic (video) colorful images. Such adaptation can be useful for an array of research topics that require the precise low-level properties of colorful visual stimuli, such as memory [7], cognitive effort [5,8,6], and social evaluation [9].

### Implementation

**Fig. 1:**
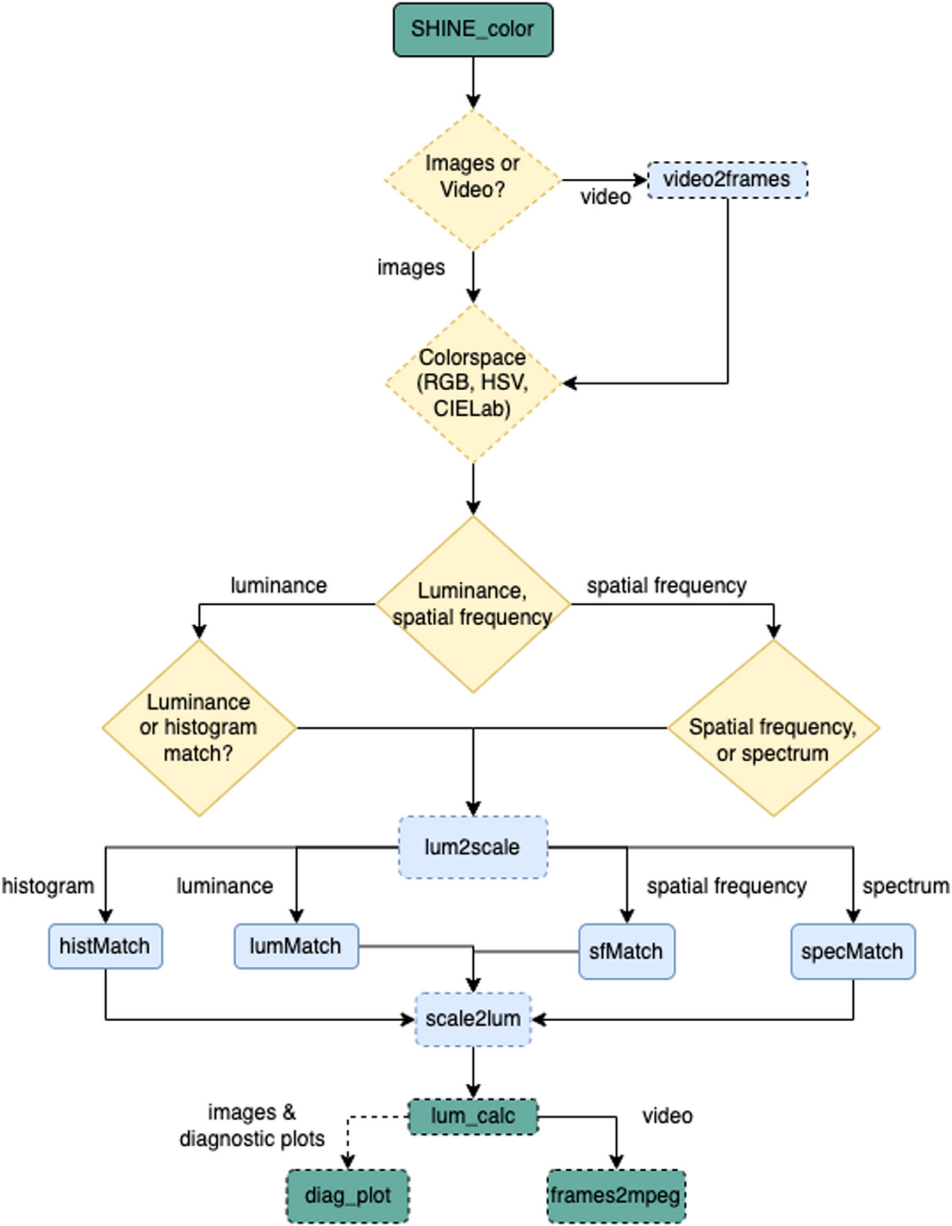
SHINE_color condensed flowchart. Functions (rounded rectangle) and decisions (diamonds) with dashed borders are introduced by SHINE_color (e.g., video2frames, lum2scale, scale2lum, lum_calc, diag_plot, frames2mpeg). They allow SHINE operations to be performed on colorful images.

The SHINE_color toolbox works in an intuitive way (complete flowchart available at OSF). The toolbox can be called directly on the command line or on MATLAB's command window. On one hand, calling the toolbox from the command line requires an advanced understanding of MATLAB logic, with the advantage of allowing users to integrate SHINE_color on analytical pipelines. On the other hand, calling the toolbox from MATALAB's command window is a user-friendly approach that allow even first-time MATLAB users to take full advantage of SHINE_color power. When calling from the command window, a wizard guides the user through a series of questions that specify the input files characteristics (either a set of images or a video), the color space to be used (i.e., HSV, CIELab, RGB), and the SHINE operations to be performed. From the user input, the toolbox performs precise image manipulations and returns manipulated images, summary statistics, diagnostic visualizations, and a log of users’ commands (Fig. 1).

All operations from SHINE are available on SHINE_color. The toolbox can precisely scale images’ luminance and contrast, specify exact histograms, and control images’ Fourier amplitude spectra, all optimized for perceptual visual quality. We strongly recommend referring to Willenbockel et al. [1] and to the SHINE user manual (http://www.mapageweb.umontreal.ca/gosselif/shine/SHINEmanual.pdf) for a detailed description of each operation.

Critically, operations can be applied directly to RGB channels or by transforming RGB to HSV or CIELab color spaces. Performing operations directly on RGB channels is preferable when equating the Fourier amplitude. Using HSV or CIELab color spaces are preferable for matching luminance and histograms without changing images’ Hue or Saturation [1]. If a video is provided, its frames are first extracted, then either RGB channels are split or RGB images are transformed to either HSV or CIELab, as per user preference. When choosing to work with RGB channels directly, operations are applied to each channel separately, one at a time (red, green, or blue) which are then combined to create the modified RGB images (see Ruedeerat [10] for a similar approach using SHINE). When choosing to work with HSV or CIELab, RGB images are transformed to one of the color spaces. The HSV color space creates Hue, Saturation, and Value (luminance) channels. Likewise, the CIELab color space creates lightness (*L*), red and green (*A*), and blue and yellow (*B*) channels. Hue and Saturation or *A* and *B* (HSV or CIELab respectively) channels are held in memory and are not manipulated. The luminance channel (either Value or *L* channel) is rescaled (from 0 to 1 or 0–100, to 0–255, HSV and CIELab respectively). Then, all operations from SHINE (Table 1) can be performed in the scaled luminance channel. For instance, displays an example of exact histogram matching. Following, the luminance channel is rescaled back to its original range and is combined with the Hue and Saturation or *A* and *B* channels. These HSV or CIELab images are then transformed back to RGB images. For videos, either working with RGB or color spaces, there is an additional step in which frames are recombined back into a video.

**Table 1:** Overview of the functions from SHINE_color. Most functions come from the SHINE toolbox, and their descriptions are also available on Willenbockel et al. [1]. Single stars (*) denotes functions that have been adapted from SHINE, double stars (**) indicates new functions from SHINE_color. Functions are listed in alphabetical order.

Function	Description
avgHist	computes average histogram
diag_plot**	creates diagnostics plots for manipulated images (luminance histogram, sfPlot, spectrumPlot)
frames2mpeg**	creates a mpeg (.mp4) video from a sequence of frames
getAllFilesInFolder**	read all frames from a folder
getRMSE	computes root mean square error
hist2list	transforms histogram into a sorted (darker-to-brigther) list
histMatch	exact histogram matching across images
imstats	computes image statistics
lum2scale**	converts RGB to HSV or CIELab color spaces, extracts the luminance channel, and scale it to grayscale range
lum_calc**	computes the luminance channel average and standard deviation
lumMatch	scales mean luminance and contrast
match	histogram specification
readImages*	read input images and apply the lum2scale function (see below)
rescale	luminance rescaling
scale2lum**	scales the luminance channel (either V channel from HSV, or L channel from CIELab) from grayscale range to HSV or CIELab range
separate	foreground-background segregation
sfMatch	equates the rotational average of the amplitude spectra
sfPlot	plots the energy at each spatial frequency
SHINE_color*	main function for loading, equating, and saving grayscale and colorful images
specMatch	matches amplitude spectrum
spectrumPlot	plots amplitude spectrum
ssim_index	computes Structural Similarity index
ssim_sens	computes SSIM gradient
tarhist	computes a target histogram
video2frames**	extracts all frames from a video

**Fig. 2:**
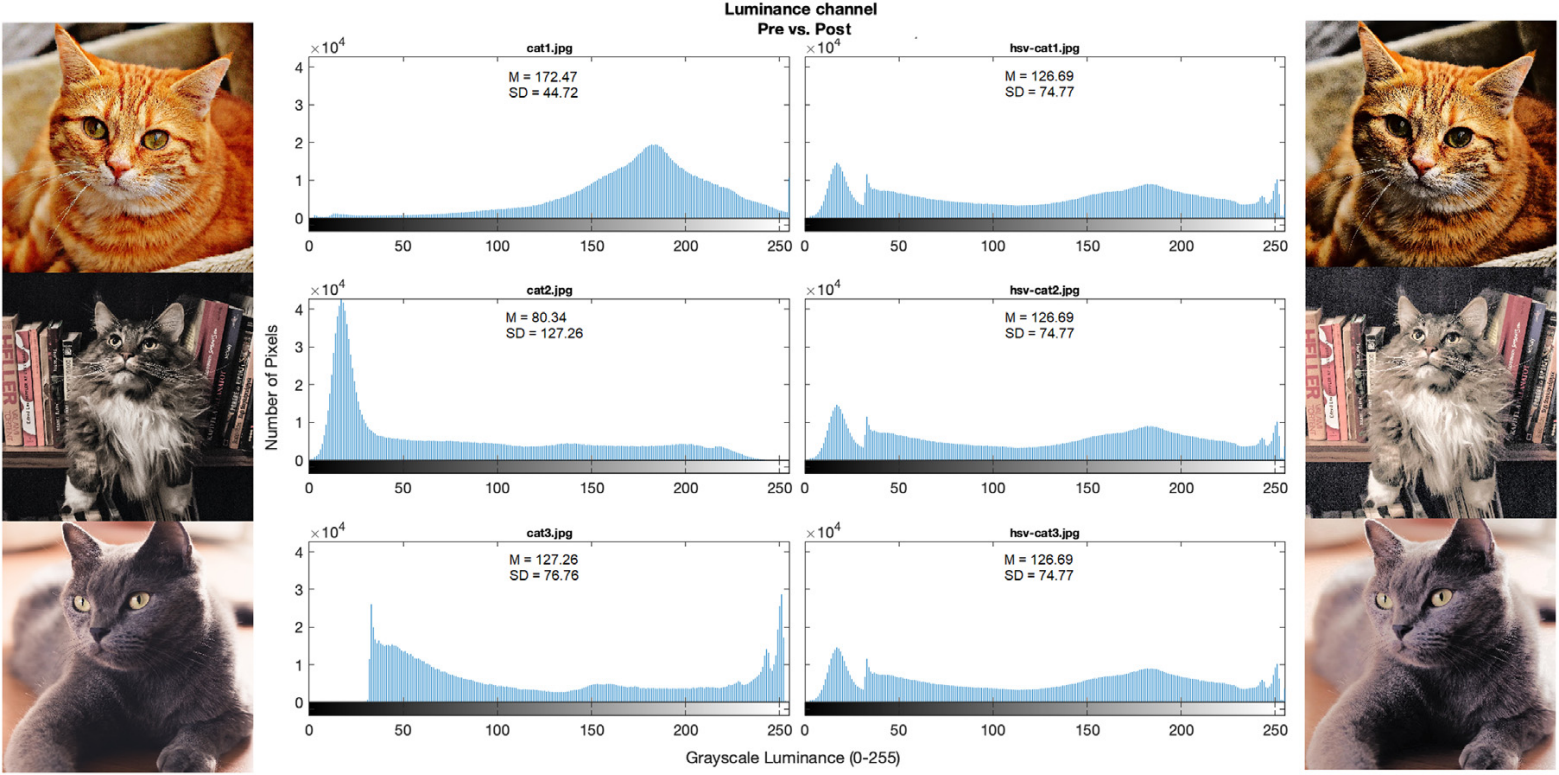
An example of the histogram matching by SHINE_color using the HSV color space. On the left there are images (from Pexels), luminance histograms, and summary statistics before the operation. On the right, we have the same elements after the operation.

SHINE_color automatically calculates the mean and standard deviation of the luminance channel before and after manipulations for both images and videos. These statistics are saved in a .txt file in the folder SHINE_color_OUTPUT/DIAGNOSTICS. In addition, for images, but not for videos, users can choose to generate diagnostic plots of luminance histogram, spatial frequency, or spectra, to compare these properties before and after manipulations (Fig. 2). When working with RGB, statistics and plots are generated for each channel. Finally, SHINE_color will automatically save a log of users’ commands in a .txt file, allowing users to review their steps across different interactions with the toolbox.

Whereas SHINE_color takes full advantage of the powerful functions from the SHINE toolbox [1] for controlling low-level properties of colorful images and videos, it is worth noting that the accurate display of SHINE_color output for experimental research ultimately depends on several factors. Of particular importance are the assumption of linearity between the manipulated luminance values and the display luminance. For instance, monitor calibration [1] and room lightning conditions [8] are essential aspects of this assumption.

The SHINE_color toolbox is openly available at OSF and GitHub. Plans for future development include a MATLAB guided user interface and an adaptation to Python language, for integration with experimental packages such as PsychoPy [11].

## Ethics statements

This work did not involve any human or animal participants.

## CRediT authorship contribution statement

**Rodrigo Dal Ben:** Conceptualization, Methodology, Software, Validation, Investigation, Writing – original draft.

## Declaration of Competing Interest

The authors declare that they have no known competing financial interests or personal relationships that could have appeared to influence the work reported in this paper.

## Data Availability

No data was used for the research described in the article.
